# Comparative Genomic Analysis of *Labrenzia aggregata* (*Alphaproteobacteria*) Strains Isolated From the Mariana Trench: Insights Into the Metabolic Potentials and Biogeochemical Functions

**DOI:** 10.3389/fmicb.2021.770370

**Published:** 2021-12-14

**Authors:** Haohui Zhong, Hao Sun, Ronghua Liu, Yuanchao Zhan, Xinyu Huang, Feng Ju, Xiao-Hua Zhang

**Affiliations:** ^1^College of Marine Life Sciences, and Frontiers Science Center for Deep Ocean Multispheres and Earth System, Ocean University of China, Qingdao, China; ^2^Laboratory for Marine Ecology and Environmental Science, Qingdao National Laboratory of Marine Science and Technology, Qingdao, China; ^3^School of Engineering, Westlake University, Hangzhou, China; ^4^Institute of Advanced Technology, Westlake Institute for Advanced Study, Hangzhou, China; ^5^Institute of Evolution & Marine Biodiversity, Ocean University of China, Qingdao, China

**Keywords:** *Labrenzia aggregata*, the Mariana Trench, sulfur cycle, nitrogen cycle, metabolic capacity, hadal zone, comparative genomics

## Abstract

Hadal zones are marine environments deeper than 6,000 m, most of which comprise oceanic trenches. Microbes thriving at such depth experience high hydrostatic pressure and low temperature. The genomic potentials of these microbes to such extreme environments are largely unknown. Here, we compare five complete genomes of bacterial strains belonging to *Labrenzia aggregata* (*Alphaproteobacteria*), including four from the Mariana Trench at depths up to 9,600 m and one reference from surface seawater of the East China Sea, to uncover the genomic potentials of this species. Genomic investigation suggests all the five strains of *L. aggregata* as participants in nitrogen and sulfur cycles, including denitrification, dissimilatory nitrate reduction to ammonium (DNRA), thiosulfate oxidation, and dimethylsulfoniopropionate (DMSP) biosynthesis and degradation. Further comparisons show that, among the five strains, 85% gene functions are similar with 96.7% of them encoded on the chromosomes, whereas the numbers of functional specific genes related to osmoregulation, antibiotic resistance, viral infection, and secondary metabolite biosynthesis are majorly contributed by the differential plasmids. A following analysis suggests the plasmidic gene numbers increase along with isolation depth and most plasmids are dissimilar among the five strains. These findings provide a better understanding of genomic potentials in the same species throughout a deep-sea water column and address the importance of externally originated plasmidic genes putatively shaped by deep-sea environment.

## Introduction

One of the largest extreme environments on Earth is the water under high hydrostatic pressure (HHP), i.e., the deep sea, which contributes up to approximately 95% of the oceans’ volume. Among the deep-sea ecosystems, hydrostatic pressure elevates greatly in oceanic trenches belonging to the hadal zone (deeper than 6,000 m). The Challenger Deep of the Mariana Trench is the deepest oceanic ecosystem on earth, in which the hydrostatic pressure elevates up to 110 MPa and the temperature remains low (1–2°C; Jamieson et al., 2010). The first obligate piezophilic microorganism isolated from the Challenger Deep was a strain MT-41 (Yayanos et al., 1981) which were classified into genus *Colwellia* later (DeLong et al., 1997). Later, many more microorganisms were recovered from the Mariana Trench thanks to developments in deep-sea sampling technologies, such as those from sediments (Kato et al., 1998; Pathom-Aree et al., 2006; Zhang et al., 2019a), animals (Kusube et al., 2017), and seawater (Zhou et al., 2018). Previous studies based on pure cultures focused on comparing piezophilic isolates from the same water depth, such as *Actinomycetes* isolates (Pathom-Aree et al., 2006) and *Shewanella benthica* (Zhang et al., 2019a), or on comparing deep-sea isolates with their counterparts from other surface waters (Peoples et al., 2020). Few studies considered the difference between the hadal and surficial isolates recovered throughout the same water column. Although recent development of non-cultivation methods (such as single cell sequencing and genome-resolved metagenomics) has greatly expanded the metabolic potentials of microbes from trenches (León-Zayas et al., 2015; Gao et al., 2019; Zhong et al., 2020), the mobile elements in genomes, such as plasmids, could not be retrieved by metagenomics (binning) because of their disproportionate sequencing depths (Maguire et al., 2020) and the structure of chromosomes and plasmids could not be observed by the highly fragmented draft genomes from these methods. Thus, an in-detail comparison based on pure cultures between vertically distributed ecotypes of the same species, especially between their mobile elements, is required to uncover the metabolic potentials and differentiation of these widely distributed bacteria in hadal environment.

*Labrenzia aggregata* (formerly *Stappia aggregata*) is heterotrophic, facultative anaerobic *Alphaproteobacteria* ubiquitous in marine environments, participating in nitrogen and sulfur cycles, such as denitrification (King, 2003; Weber and King, 2007; Wyman et al., 2013) and dimethylsulfoniopropionate (DMSP) production (Curson et al., 2017). DMSP is an important osmoprotectant (Cosquer et al., 1999; Kiene et al., 2000) or a carbon source (Yoch, 2002; Curson et al., 2011; Zhang et al., 2019b) for bacteria. *L. aggregata* has also been reported involving in carbon monoxide oxidation (Weber and King, 2007). Furthermore, an ammonia monooxygenase gene was predicted related to nitrification in the type strain *L. aggregata* IAM 12614 (Coates and Wyman, 2017). Besides, there was a speculation of carbon fixation *via* the Calvin cycle in *L. aggregata* considering the existence of the key enzyme ribulose-1,5-bisphosphate carboxylase-oxygenase (RuBisCO; Weber and King, 2007). Due to the incompleteness of the previously reconstructed genomes, these predictions require further examination.

In this study, we isolated four strains of *L. aggregata* at the Challenger Deep from surface water down to 9,600 m depth. Another *L. aggregata* strain LZB033 with a draft genome assembly isolated previously from the surface water of East China Sea (Curson et al., 2017) was also included for comparison. Complete genomes of the five strains were sequenced and assembled, resulting in more detailed chromosome-plasmid level insights into functional prediction of this species and the locations of the key genes. Eventually, we draw an overview of the metabolic potentials on how *L. aggregata* differs between the surficial and hadal strains and survives in the deepest ocean.

## Materials and Methods

### Sampling, Isolation, Cultivation, and DNA Extraction

All seawater samples were collected using Niskin bottles. SDL044 was isolated from the surface seawater samples (25° 0.527′N, 145° 59.514′E) of the Mariana Trench in October 2017. RF14 was isolated from the seawater samples (11° 23.036′N, 142° 30′E) of the Mariana Trench at a depth of 4,000 m in April 2016. ZYF703 and ZYF612 were isolated from seawater samples (11° 22.160′N, 142° 20.524′E) of the Mariana Trench in November 2016 at depths of 9,600 m. All these four strains were routinely grown on marine agar 2216E (MA; Becton Dickinson) at 28°C. A single colony was picked from original plates, and streaked and re-streaked at least three times to ensure purification (Zhao et al., 2020). Genomic DNA was extracted following the phenol-chloroform-isoamylic alcohol extraction protocol of Marmur (1961). For the pre-test of cultivation under HHP, liquid media inoculated with the cultures were diluted into the same optical densities as above. Next, these media were aspirated into 2.5 ml syringes before transfer into high pressure reactors for 2 weeks at 40, 60, and 80 MPa, 28°C as shown in Zheng et al. (2020). In total, there were three syringes per strain (triplicates) per pressure. After that, the media in the 1x, 10x, and 100x dilutions were cultivated on MA using the spread plate technique for 2 days at 28°C, and subsequently, the average colony numbers were counted and calculated. Growth curves of these five strains under different temperatures (4°C, 16°C, 28°C, and 37°C) were recorded by measuring the optical densities under atmospheric pressure.

### Sequencing and Assembling

All the five strains were sequenced by Illumina Hiseq 4,000 with a 270 bp pair-end library and PacBio with 20 kb library at the Beijing Genomics Institute (BGI; Shenzhen, China). Quality control of reads was performed at the same institute. As for assembling, firstly, the PacBio reads were assembled with canu (v1.3; Koren et al., 2017), and Illumina reads were used later as correction by REAPR (v1.0.18; Hunt et al., 2013). Settings in assembling were all default. Later, the Illumina reads were mapped back to the assembled genomes with BBMap (Bushnell, 2014) to calculate the sequencing depths, which were mapped read numbers multiplying by read length then divided by the total length of the chromosome or plasmid.

### Genomic Annotation and Analyses

Annotations of these genomes were conducted by RASTtk online service with default settings (Brettin et al., 2015) and most of the analyses (including CDS numbers and gene count) are based on this result. The genomes were also annotated by eggnog-mapper on EggNOG database v5.0 (Huerta-Cepas et al., 2019) to eliminate suspicious results. Ambiguous annotations were manually checked against the KEGG database and non-redundant (NR) database in NCBI. Further clustering of 95% identical genes was performed by CD-HIT v 4.8.1 (Fu et al., 2012) with parameters “-c 0.95 -g 1.”

For the phylogenomic analysis of the five strains, the DNA sequences encoding the 52 ribosomal proteins were selected and aligned individually by a codon-specific multiple-sequence aligner MACSE (Ranwez et al., 2018) with parameters “-gc_def 11.” Phylogenetic inference was conducted by IQ-TREE v 2.1.3 (Minh et al., 2020) based on a manually concatenation of the ribosomal protein genes with parameters “-B 1000 -m MFP --seqtype CODON11,” which could automatically select the best-fit codon model according to Bayesian information criterion. ANIs between these strains were calculated by JspeciesWS (Richter et al., 2016). For the phylogeny of *rbcL* genes, all of Swiss-Prot reviewed reference genes were downloaded from UniProt with key word rbcL/RuBisCO or rpl/RuBisCO-like proteins. After downloading, these amino-acid sequences were de-replicated with an 80% identity cutoff by CD-HIT as mentioned above with parameter “-c 0.8.” Then, the amino-acid sequences of the *rbcL* genes from *L. aggregata* and reference were aligned by MAFFT v7.471 with default parameters (Katoh and Standley, 2013). After that, we deleted the sites that contains gaps in more than 50% sequences from the alignment. Finally, phylogenetic analysis was conducted by IQ-TREE 2 based on the curated alignment with parameters “-B 1000 -m MFP --msub nuclear”.

Prophages in the genomes were detected by PHASTER with default settings (Arndt et al., 2016). To identify the genes encoding secondary metabolites and antibiotic resistance genes (ARGs), the genes were firstly searched against the structured antibiotic resistance gene database (SARGs) as described by Yang et al. (2013). The annotation of candidate ARGs was further confirmed by comparing with those derived from the aforementioned RAST, EggNOG, and KEGG database to eliminate ambiguous assignment. The ARGs were further manually assigned into resistance types and subtypes (Yang et al., 2016). Biosynthetic gene clusters of secondary metabolites, including NRPS, were mined by the antiSMASH database and tools with default settings (Blin et al., 2019).

### Disk Diffusion Tests

Disks with diameters of 6 mm containing the same concentration of antibiotics were put on seeded agar plates. The concentration of the five strains was adjusted to the same (the optical density) before the test to make sure the results were comparable. The plates were cultured for 24 h at 28°C before the diameters of the inhibition zones were documented for downstream analysis.

## Results and Discussion

### Phylogenomic Analysis Confirms That the Five Strains Belong to the Same Species

**Figure 1:**
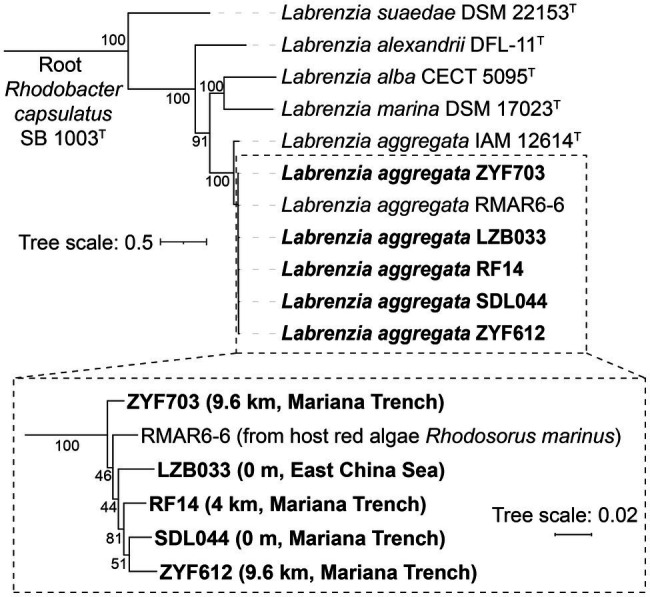
Phylogenetic inference of the *Labrenzia aggregata* strains. This is a maximum-likelihood codon tree in KOSI07 + F + R3 model selected by ModelFinder in IQ-TREE 2 (Minh et al., 2020) based on concatenated coding sequences of 52 ribosomal proteins with a total of 22,932 nucleobases. This tree was tested by 1,000 replicates of ultra-fast bootstrap method, and the percentage of support for each branch is labeled near the node.

Four *L. aggregata* strains SDL044, RF14, ZYF703, and ZYF612 were isolated from seawater samples from the Mariana Trench at depths of 0 m, 4,000 m, 9,600 m, and 9,600 m, respectively. Since each of these strains contains three copies of 16S rRNA genes and resolution of these genes is not high enough between strains, 52 ribosomal protein genes, which encode some of the most conserved proteins in all lives and are commonly used for phylogenomic analysis, were chosen for their classification, including those from *L. aggregata* strains and several reference genomes (Figure 1). In addition to LZB033, all the five strains are clustered on the same branch in the tree and located distantly from other species, indicating they all belong to *L. aggregata* with 100% branch support. Moreover, average nucleotide identities (ANIs) between each of the strains and the representative genome of *L. aggregata* are between 97 and 98%, which is higher than the empirical intra-species threshold – 95% (Goris et al., 2007; Supplementary Table S1), furtherly confirming the classification of the four isolates from the Mariana Trench.

### Geographical Origin of the Hadal Strains

A pressurization test shows all the four strains from the Mariana Trench could partially tolerate HHP with higher survival rate in the hadal strains (Supplementary Figure S1). Another cultivation experiments measured by optical densities suggest that under atmospheric pressure (0.1 MPa), all the five strains grow optimally under 37°C (Supplementary Figure S2). We could not observe any noticeable change in optical densities of these strains during a cultivation under 4°C, 80 MPa for two weeks. Thus, all these results indicate that the deep-sea *L. aggregata* strains are not piezophilic and they have not completely adapted to hadal HHP. It is more likely that the two hadal strains sank from surficial water recently and were inactive (or grew extremely slowly) under HHP at 9,600 m depth. Previously reported non-piezophilic trench species *Pseudomonas bathycetes*, which was the first isolate from sediments of the Mariana Trench, grew slowly with generation time up to 33 days under *in situ* condition: 2°C, 100 MPa (Schwarz and Colwell, 1975). Several recent studies have shown the connectivity of deep-sea and surficial microbial communities and most of the dominant prokaryotes from the deep ocean can also be detected in surface waters according to operational taxonomic unit analysis (Agustí et al., 2015; Mestre et al., 2018). Our isolations could be living examples of these sinking microbes.

### Genomic Composition: Similar Core Chromosomic Genes but Differential Plasmids

**Figure 2:**
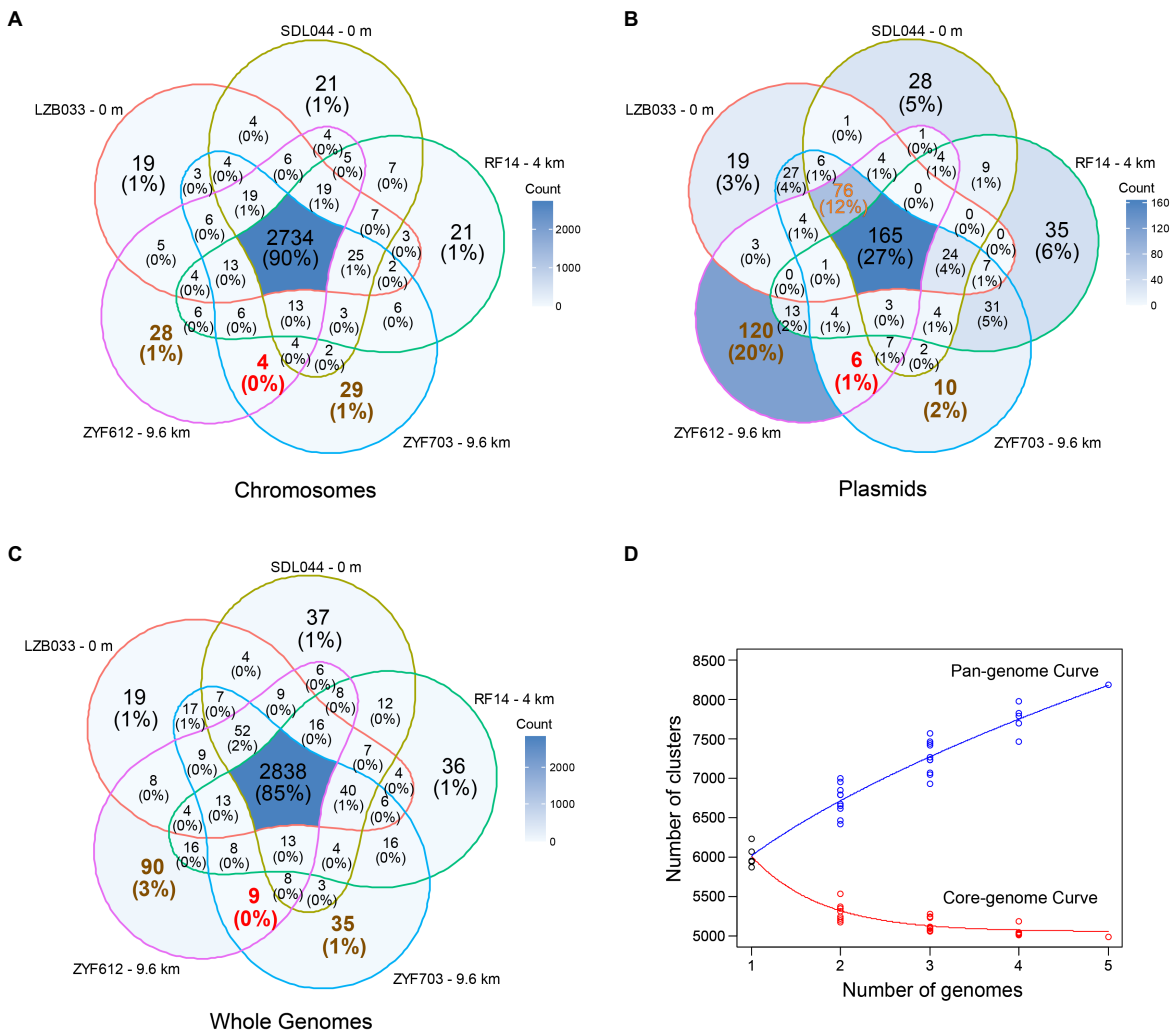
Venn diagrams of the gene function numbers according to RAST annotations (Brettin et al., 2015) in the **(A)** chromosomes, **(B)** plasmids, and **(C)** whole genomes of the five *L. aggregata* strains. Genes with the same annotation are counted only once regardless of their identities or coverages. Hypothetical protein genes are excluded. The percentage of each part is labeled under each count. Chromosomic genes are functionally highly similar among the five strains, while most functional specific genes are contributed by the plasmids. **(D)** Predicted curve fittings of pan-genome and core-genome of the five strains according to 95% nucleotide identity gene clustering by CD-HIT (Fu et al., 2012) including hypothetical proteins.

Each of the five strains consists of one large chromosome (5.83 to 5.89 Mbp) and 2–4 plasmids (Table 1). These plasmids and chromosomes are distinguished by their sizes and differential sequencing depths (Supplementary Table S2). To avoid ambiguity, the plasmids are sorted and named by their sizes (Table 1, P1 ~ P4). All of these genomes share similar GC contents of 59.0 ± 0.2% and total sizes of 6.49–6.96 Mbp, whereas the strains from greater depths have larger total size of plasmids (the largest one is 76.6% larger than the smallest one). With these genomes getting functionally annotated, more than 6,000 coding DNA sequences (CDSs) are predicted in each of these strains. Consistent with the trends of genome size, the greater the depth from which the strain was recovered, the more CDSs are predicted; the 9.6 km strain ZYF612 contains 8.12% more CDSs (predominantly contributed by the plasmids) than the 0 m strain SDL044. When removing the redundancy of the annotation, the gene function numbers of the five strains differ slightly, ranging from 3,048 to 3,121, which are inconsistent with their sizes: the 4 km strain RF14 (third largest in size) contains only 3,048 annotated gene functions. Examinations based on Venn diagrams (Figures 2A–C) suggest 90% gene annotations on the chromosomes are similar, while only 27% are shared by the plasmids. The 9.6 km strain ZYF612 harbors most specific genes majorly contributed by its plasmids (120 or 20% of total plasmidic gene functions, Figure 2B). Since the ZYF612 contains an extra of more than 400 CDSs than other strains (6,825 vs. 6,248 ~ 6,456) but similar gene function numbers (3,121 vs. 3,048 ~ 3,088), these extra genes should mainly from multiple copies and hypothetical protein genes (more than 12.6%; 2,215 vs. 1,863 ~ 1,967). Further investigation suggests most of these multiple copies are related to phages and thus, these gene duplications might be a sign of phage infections in ZYF612. Details of the phage-related genes will be discussed in a following section “Horizontal Gene Transfers in *L. aggregata*”. Another analysis based on 95% nucleotide identity gene clustering (Figure 2D) suggests the pan-genome of *L. aggregata* could still be fast-expanding if adding more strains while the core-genome is relatively stable.

**Table 1:** Genomic information of the five completely sequenced *Labrenzia aggregata* strains.

Strain	Isolation Location	Isolation Depth (m)	Isolation Source	Genome (Mbp)	Chromosome (Mbp)	Plasmids Total (Mbp)	Each Plasmid (Mbp)	G + C (%)	CDS	Unique Gene Annotations	Hypothetical Proteins	tRNA
LZB033	East China Sea	0	Water	6.49	5.85	0.64	0.45, 0.19	59	6,248	3,052	1,863	52
SDL044	Mariana Trench	0	Water	6.55	5.89	0.66	0.46, 0.10, 0.07, 0.04	59.1	6,312	3,067	1,913	53
RF14	Mariana Trench	4,000	Water	6.62	5.87	0.75	0.27, 0.25, 0.13, 0.10	59	6,421	3,048	1,952	52
ZYF703	Mariana Trench	9,600	Water	6.69	5.88	0.81	0.45, 0.19, 0.17	59	6,456	3,088	1,967	51
ZYF612	Mariana Trench	9,600	Water	6.96	5.83	1.13	0.47, 0.25, 0.22, 0.19	58.8	6,825	3,121	2,215	54

The plasmids are sorted in descending order of their sizes and named after this order herein (LZB033P1, LZB033P2; SDL044P1 ~ SDL044P4; RF14P1 ~ RF14P4; ZYF703P1 ~ ZYF703P3; ZYF612P1 ~ ZYF612P4; and P1 = largest in size).

### Metabolic Reconstruction: Core Carbon Metabolism

**Figure 3:**
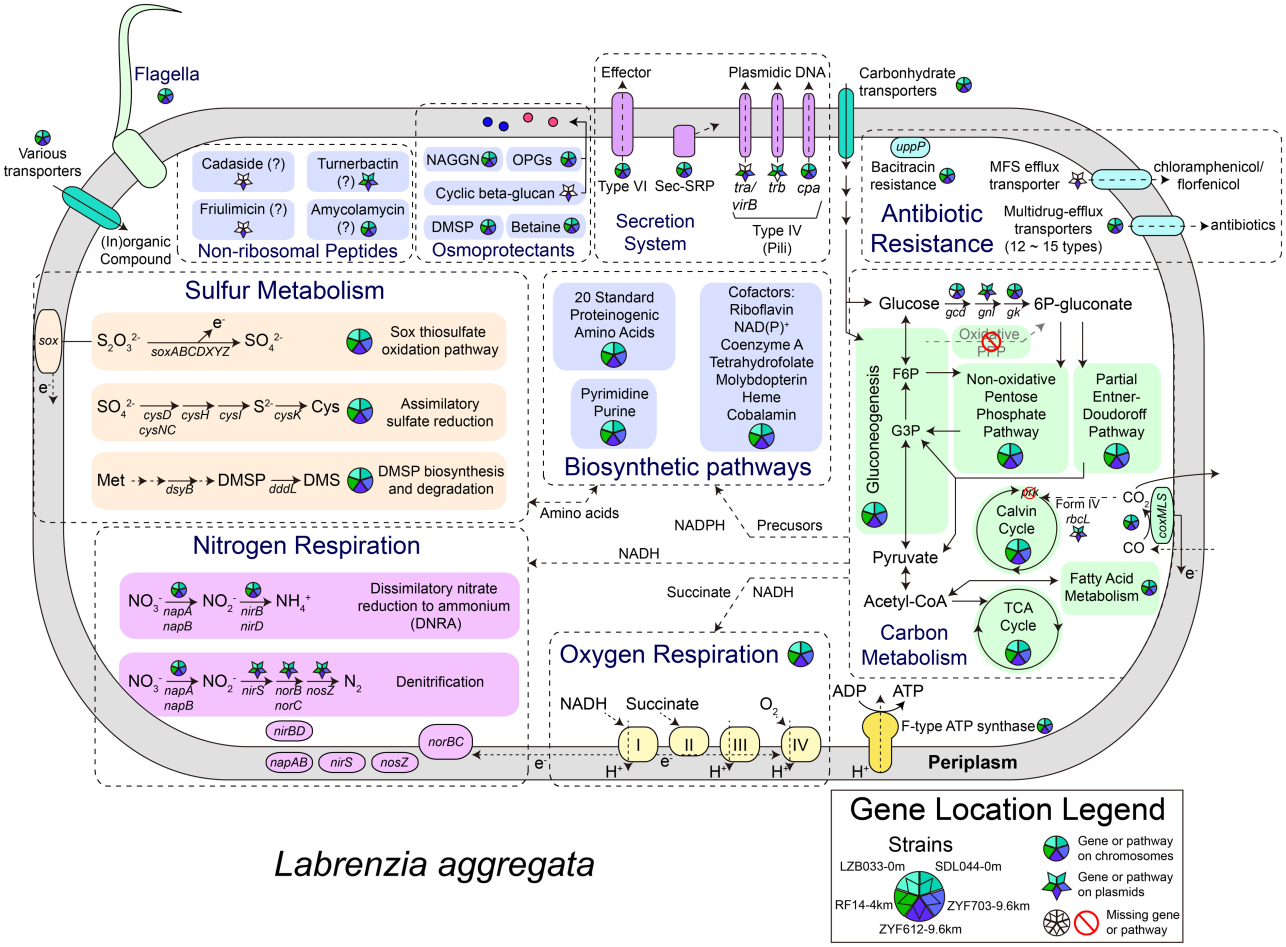
Metabolic reconstruction of *L. aggregata*. The gene locations and presence in the five strains are depicted as divided circles and pentagrams. G3P, glyceraldehyde 3-phosphate; F6P, fructose 6-phosphate; 6P-gluconate, 6-phospho-gluconate; TCA, tricarboxylic acid; PPP, pentose phosphate pathway; DMSP, dimethylsulfoniopropionate; DMS, dimethyl sulfide; OPGs, osmoregulated periplasmic glucans; and NAGGN, N-acetylglutaminylglutamine amide.

As shown in their metabolic reconstruction maps (Figure 3), the core metabolisms of the five strains are highly similar. Gluconeogenesis (GNG) and non-oxidative pentose phosphate pathway (PPP) are predicted in all of them. All the five genomes are devoid of the key gene encoding 6-phosphofructokinase in glycolysis (Embden–Meyerhof–Parnas pathway) or the gene encoding 6-phosphogluconolactonase participating in oxidative PPP and classical Entner-Doudoroff (ED) pathway (Ahmed et al., 2005), making these strains incapable of degrading glucose through these canonical pathways. Instead, a modified pathway incorporating glucose dehydrogenase (*gcd*), gluconolactonase (*gnl*), and gluconokinase (*gk*) is encoded in four strains except for RF14, generating 6-phospho-gluconate for subsequent non-oxidative PPP or the latter half of ED pathway. Similar modified glucose degrading pathways were reported previously in several *Cyanobacteria* (Chen et al., 2016). The genes *gcd* and *gnl* also involve in the archaeal semi-phosphorylative and non-phosphorylative ED pathway (Lamble et al., 2005; Reher and Schönheit, 2006), but a following key enzyme gluconate dehydratase is absent in all the five strains, making the modified ED pathway in *L. aggregata* different from these variants. Interestingly, only *gnl* genes are encoded on the largest plasmid of each strain (referred to as P1 hereafter, including LZB033P1, SDL044P1, RF14P1, ZYF703P1, and ZYF612P1), while all other genes are on the chromosomes. Overall, genetic predictions suggest that at least four strains (except for RF14) could conserve energy through complete glucose (and related carbohydrates) degradation into CO_2_ *via* the modified ED pathway, the tricarboxylic acid cycle, and the respiratory chain.

CO oxidation is predicted in all the five strains according to the presence of aerobic carbon monoxide dehydrogenase complex (*coxL, coxM, and coxS*). The generated CO_2_ was previously speculated being assimilated by the autotrophic Calvin cycle in the type strain of *L. aggregata* considering the presence of the gene *rbcL* (also known as *cbbL*, encoding the key enzyme RuBisCO; King and Weber, 2007). However, a phylogenetic analysis of the *rbcL* (Supplementary Figure S3) indicates that these *rbcL* genes encode form IV RuBisCO proteins, also known as RuBisCO-like proteins, which are incapable of catalyzing CO_2_ fixation (Tabita et al., 2008); another gene encoding phosphoribulokinase (*prk*) essential in the Calvin cycle is also absent in all the five strains. In addition to the absence of other complete carbon fixation pathways, these five strains therefore are unlikely mixotrophic or capable of carbon fixation. The CO oxidation in *L. aggregata* might only function as a supplementary source of electrons for the respiratory chain, and the CO_2_ molecules generated in this process are more likely being released instead of being assimilated. CO oxidation in deep-sea microbes (putatively in *Bacteroidetes* or *Chloroflexi*) has been reported previously in the Mediterranean (Martin-Cuadrado et al., 2009) and our isolates represent an *alphaproteobacterial* origin of these genes in deep sea.

### Genes Related to Respiration and Biogeochemical Cycles

An electron transport chain is encoded in each genome of strains with oxygen or nitrate as final electron acceptors. Under anoxic conditions, genetically nitrate could be reduced into nitrogen (denitrification) or ammonium (DNRA) according to the presence of dissimilatory periplasmic nitrate reductase (*napA and napB*), nitrite reductase (NO-forming; *nirS*), nitric oxide reductase (*norB and norC*), nitrous oxide reductase (*nosZ*), and NADH-dependent nitrite reductase (*nirB and nirD*). All these genes have been detected in *L. aggregata* type strain IAM 12614 previously (Coates and Wyman, 2017), and denitrification in *L. aggregata* was also reported with experimental evidence (Weber and King, 2007). Interestingly, the denitrification genes are all located on the largest plasmid of each strain (P1), while the DNRA genes are encoded on chromosomes, indicating they might have different origins. Also an extra copy of *nosZ* and related genes (operon *nosRZDFYL*) could be predicted on the plasmid ZYF612P2. Another previously reported gene encoding ammonia monooxygenase related to nitrogen cycle (nitrification) in the strain IAM 12614 (Coates and Wyman, 2017) is absent in all our five strains.

When it comes to sulfur metabolism, all the four Mariana Trench strains contain the key DMSP biosynthetic gene *dsyB*, >99% amino-acid identical to the experimentally confirmed one in LZB033 (Curson et al., 2017). The gene for DMSP cleavage (*dddL*) is also universal among them, both suggesting a common role for *L. aggregata* in DMSP metabolism throughout the ocean. Other sulfur-related genes include a complete *sox* thiosulfate oxidation pathway that is encoded in each of the strains, possibly serving as a supplementary source of electrons for ATP biosynthesis. Moreover, genetically sulfate could be reduced for (homo-)cysteine biosynthesis (assimilatory sulfate reduction) in the five strains according to the presence of related genes.

### Specific Genes in Hadal Strains

According to the genome annotations, only nine gene functions are shared by the two hadal strains and are absent in other strains (as shown in red fonts in Figure 2C). There are more strain-specific gene functions in the two hadal strains (90 in ZYF612 and 35 in ZYF703, shown in brown fonts in Figure 2C). The functions of these genes could not be summarized by one or two pathways and are divided into various functional categories, some of which related to hadal environment will be discussed separately in the following paragraphs.

Bacteria adapt to HHP through various changes, such as osmoregulation and respiratory chain (Simonato et al., 2006), the former of which relies on organic osmolytes or osmoprotectants (Galinski, 1995; Wood, 2011). Biosynthetic pathways of osmoprotectants in the five *L. aggregata* strains include DMSP, betaine (trimethylglycine), N-acetylglutaminylglutamine amide (NAGGN), and osmoregulated periplasmic glucans (OPGs). DMSP has been discussed in the previous section. All the five strains encode two distinct betaine synthesis pathways: oxidation of choline (Cánovas et al., 1996) and methylation of glycine (Kerovuo et al., 2000). The previously identified NAGGN biosynthetic genes (Sagot et al., 2010) are also shared by all the strains. OPGs are located in the periplasm of Proteobacteria and are essential components in extreme environments (Bohin, 2000). Most of the OPG genes are shared by the five strains on chromosomes, whereas a giant gene encodes the synthetase for a unique OPG, i.e., cyclic beta-1,2-glucan, which is predicted exclusively in the plasmid of the strain ZYF612 (Supplementary Figure S4). Cyclic beta-1,2-glucan is previously found in other *Alphaproteobacteria*, such as *Agrobacterium, Rhizobium*, and *Brucella* as an osmolyte or virulence factor (Dylan et al., 1990; Arellano-Reynoso et al., 2005). In ZYF612, this gene is most similar to that from *Labrenzia suaedae* (93% amino-acid identity) isolated from a plant, *Suaeda maritima* that grows on tidal flats (Bibi et al., 2014). As for respiratory chain, in the 9.6 km strain ZYF612, several genes encoding *ccb3* cytochrome c oxidase, which are essential to the respiratory chain, are multiple copies located both on the chromosome and the plasmids with identities smaller than 95%, indicating different origins of these genes. Previous studies also found multiple clusters of such oxidase genes in piezophilic bacteria *Photobacterium profundum* SS9 (Vezzi et al., 2005) and *Shewanella benthica* (Zhang et al., 2019a). Although the hadal *L. aggregata* strains have not completely adapt to hadal environment, these genomic features may help them survive under the extremely HHP at 9,600 m depth.

Secondary metabolites, such as antibiotics, may give hadal microbes advantages when competing with other microbes, especially in the trenches where nutrients are accumulated by the physical topography (Jamieson et al., 2010) leading to more heterotrophic competitors. Several giant gene clusters encoding non-ribosomal peptide synthetases (NRPSs) were detected (Supplementary Figure S5) in the *L. aggregata* strains. These giant multi-domain enzymes synthesize peptide secondary metabolites without ribosomes or mRNA, and some of these metabolites are important antibiotics, antifungal, and antiparasitic agents (Cane and Walsh, 1999; Koglin and Walsh, 2009). Two NRPS gene clusters are present in each of the five strains and located on the chromosome and the largest plasmid of each strain (P1), while three other NRPS gene clusters are only predicted on the smallest plasmid of ZYF612 (ZYF612P4). According to functional prediction, the shared plasmidic NRPS might synthesize a siderophore involving in iron transport (turnerbactin-like, 30% of genes show similarity according to the default identity threshold in antiSMASH v5.0, Blin et al., 2019), while the shared chromosomic NRPS is suspiciously an antibiotic synthase (amycolamycin-like, only 2% of genes show similarity). The specific NRPS gene clusters in ZYF612 are all predicted to be antibiotic-related but their identities with other known clusters are relatively low (approximately 9% of genes show similarity, Supplementary Figure S5). These giant genes (such as the NRPS and cyclic beta-1,2-glucan synthase genes) demand a great amount of energy and amino-acid precursors (Reva and Tümmler, 2008). Although functions of these giant genes may be predicted tentatively, their existence may well be crucial for the survival of ZYF612 in the deepest water even with such high costs.

Apart from antibiotic biosynthesis, antibiotic resistance genes also differ in some hadal strains. In the four Mariana Trench strains, the antibiotic resistance gene (ARG) numbers (>50% identities against SARG database) range from 6 to 10, while the ZYF612 strain contains the most. A chloramphenicol/florfenicol resistance gene is specifically predicted in the plasmid ZYF612P1. Furthermore, a simple antibiotics disk diffusion test confirms the higher resistance against chloramphenicol in the three deep-sea strains than the surficial strain SDL044, while the two hadal strains are more resistant to quinolones (Supplementary Figure S6). Although currently there is no strong evidence of high antibiotic concentration in the Mariana Trench, the antibiotic resistance genes might be an advantage competing against other bacteria.

### Horizontal Gene Transfers in *L. aggregata*

**Figure 4:**
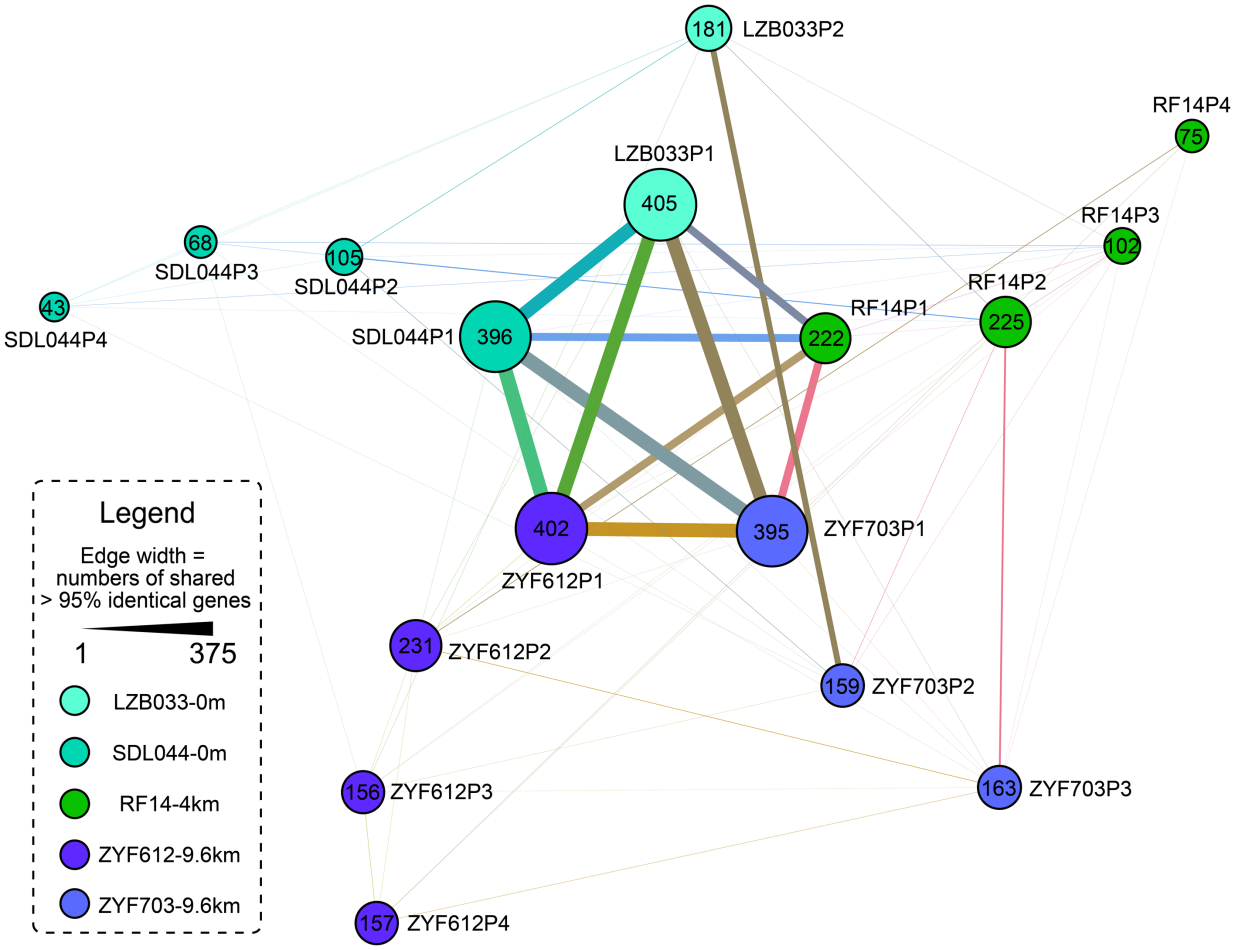
Network of the plasmidic identical genes (considering nucleotide identity >95% as identical) in the five *L. aggregata* strains. Each node represents a plasmid and the size of it reflects the numbers of genes after removing multiple copies. The widths of the edges indicate the numbers of shared genes. Different from Figure 2B, this is a network based on identity regardless of annotation; thus, the results also consider previously ignored hypothetical proteins. Most genes are shared by the longest plasmids of the five strains and the other plasmids encode more specific genes. The plasmids are named after their lengths as mentioned in Table 1. All these plasmids encode less than 20 identical genes with the chromosomes (mainly in P1; data not shown).

As discussed in the previous sections, several genes are located in the plasmids, such as the denitrification genes. Plasmids could be transferred horizontally between strains by bacterial conjugation *via* pili, which belong to the type IV secretion system (Llosa et al., 2002; Kline et al., 2010; Maier and Wong, 2015). Thus, these plasmidic genes are more tend to be external origin than the chromosomic genes. At least three types of putative pili genes are predicted in the five strains, with one *cpa* type ubiquitously in the chromosomes and two other types uniquely on the plasmids of some strains (Figure 3). Since the annotation-based Venn diagram (Figure 2B) is not enough for uncharacterized (hypothetical) proteins, an identity-based clustering of the plasmidic genes is conducted, and the result (Figure 4) suggests the largest plasmid of each strain (P1) is relatively conserved while the other plasmids differ greatly. It is tempting to speculate that the P1 plasmids in all the five strains are a stable component of the genome while all other plasmids are more frequently transferred horizontally. Since the size of the plasmid RF14P1 is smaller than the others (0.27 Mbp vs. 0.45–0.47 Mbp, Table 1) and the shared genes are likewise fewer (shown as the narrower edges in Figure 4), it is likely that the plasmid RF14P1 experienced several gene deletion, resulting in the missing 76 gene functions that are shared in the other four strains (shown in orange fonts in Figure 2B), such as the gene *rbcL* mentioned above. Gene acquirements of the 76 genes independently in the four strains are unlikely because these shared genes are highly similar (> 95% identity as shown in the edges of Figure 4).

A gene origin analysis based on gene annotations (Supplementary Table S3) suggests that the extra genes in the hadal strains predominantly come from species in *Bradyrhizobiaceae*, *Oceanicola*, *Sphingomonadales* (all belong to *Alphaproteobacteria*), and *Actinobacteria*. But the numbers of these annotated extra genes in ZYF612 (totally 94 genes) are much less than the extra 400 CDSs. It is reasonable to consider functionally similar proteins with different origins or hypothetical proteins as other contributors of the difference between the annotation-based and identity-based results (Figures 2B, 4). For example, in ZYF612, the copy numbers of genes of external origin, such as phage-associated and transposase genes, are much higher than those in other strains. In the plasmids of ZYF612, there are 66 copies of mobile element proteins (with domains similar to transposases), higher than the average copy number of these in the other four strains (16 copies), which could partially explain why ZYF612 harbors 400 more CDSs than other strains while the gene function number varies slightly. A previous study has also reported transposase being the most overrepresented COG categories in deep-sea microbial communities by fosmid clones (DeLong et al., 2006). As expected, two intact prophages could be predicted on the chromosomes of the two hadal strains ZYF703 and ZYF612 and other two intact ones on the plasmid ZYF612P2 (Supplementary Table S4). More incomplete or questionable prophages are also predicted in all the three deep-sea strains including RF14 (Supplementary Table S4). These prophages could be another origin of the larger size of the three deep-sea strains; for example, all the prophage proteins contribute to at least 0.11 Mbp genome size and 133 CDSs in ZYF612. Previous studies also reported phage insertion in *Geobacillus* genomes from the Mariana Trench (Takami et al., 2004) and in *Colwellia* genomes from various locations including the Mariana Trench (Peoples et al., 2020). Raised virus prokaryote ratio in hadal water compared to that in surface water has also been reported in the Mariana Trench (raised from 10.8-fold to 41.1-fold, Nunoura et al., 2015) and the Japan Trench (raised from 20 ~ 50-folds to 80 ~ 260-fold, Nunoura et al., 2016). Therefore, it could be postulated that HGT by virus may be another important source of external genes and partially shape the pan-genome of hadal species.

Overall, external genes were acquired by the hadal strains to confront the hostile environment in trenches, such as the higher hydrostatic pressure. Compared to our recently report of microorganisms that thrive in the hadal zone by consuming hydrocarbons (Liu et al., 2019), the hadal strains of *L. aggregata* have not changed their core metabolism greatly and still retain similar catabolic potentials like their counterparts from shallower depths. Unlike obligate piezophiles, these deep-sea ecotypes sinking from surface water still reserve many genetic potentials to survive in various other environments.

## Conclusion

The completely sequenced genomes reveal how *L. aggregata* strains may change to resist higher hydrostatic pressure. Genomically, almost identical chromosomes but dissimilar plasmidic composition suggest extra genetic potentials are horizontally acquired partially by phages and mainly through plasmid exchanges (conjugation), such as those large genes encoding cyclic beta-1,2-glucan synthase and NRPS on ZYF612’s plasmids. Genes involving in biogeochemical cycles, such as the denitrification genes, may also be overlooked by binning in certain lineages due to their plasmidic locations. Comparisons of these five strains offer a vertical genomic view of a microbial species along the seawater column, which show that instead of one single common solution, many different strategies have been evolved by the microorganisms concurrently to confront hostile deep-sea environments. In conclusion, our findings provide a metabolic framework for future studies of *L. aggregata*, and moreover, a template of ubiquitously distributed marine bacteria throughout oceans.

## Data Availability Statement

The five Labrenzia genome assemblies were uploaded onto NCBI RefSeq with the following accession numbers: GCF_001932055.2 for LZB033, GCF_020882605.1 for SDL044, GCF_020882585.1 for RF14, GCF_020882625.1 for ZYF703 and GCF_020882645.1 for ZYF612.

## Author Contributions

X-HZ designed the experiments, analyzed the data, and wrote the manuscript. HZ performed the experiments, analyzed the data, prepared all figures, and wrote the manuscript. HS performed the DNA extraction and the genome analysis. RL performed the phenotypic experiments and data analysis. YZ performed the bacterial phage analysis. XH and FJ performed antibiotic resistance analysis. All authors edited and approved the manuscript.

## Funding

This work was funded by the National Natural Science Foundation of China (91751202 and 41730530), the National Key Research and Development Program of China (2018YFC0310701), and the Fundamental Research Funds for the Central Universities (202172002).

## Conflict of Interest

The authors declare that the research was conducted in the absence of any commercial or financial relationships that could be construed as a potential conflict of interest.

## Publisher’s Note

All claims expressed in this article are solely those of the authors and do not necessarily represent those of their affiliated organizations, or those of the publisher, the editors and the reviewers. Any product that may be evaluated in this article, or claim that may be made by its manufacturer, is not guaranteed or endorsed by the publisher.
