# Supplementary material for: Comparative Genomic Analysis of Labrenzia aggregata (Alphaproteobacteria) Strains Isolated From the Mariana Trench: Insights Into the Metabolic Potentials and Biogeochemical Functions
Source: Front Microbiol. 2021 Dec 14;12:770370. doi: 10.3389/fmicb.2021.770370 (PMC8712697; doi:10.3389/fmicb.2021.770370)
Supplement: Supplementary file 1 [file Data_Sheet_1.docx]

**Supplementary Material**


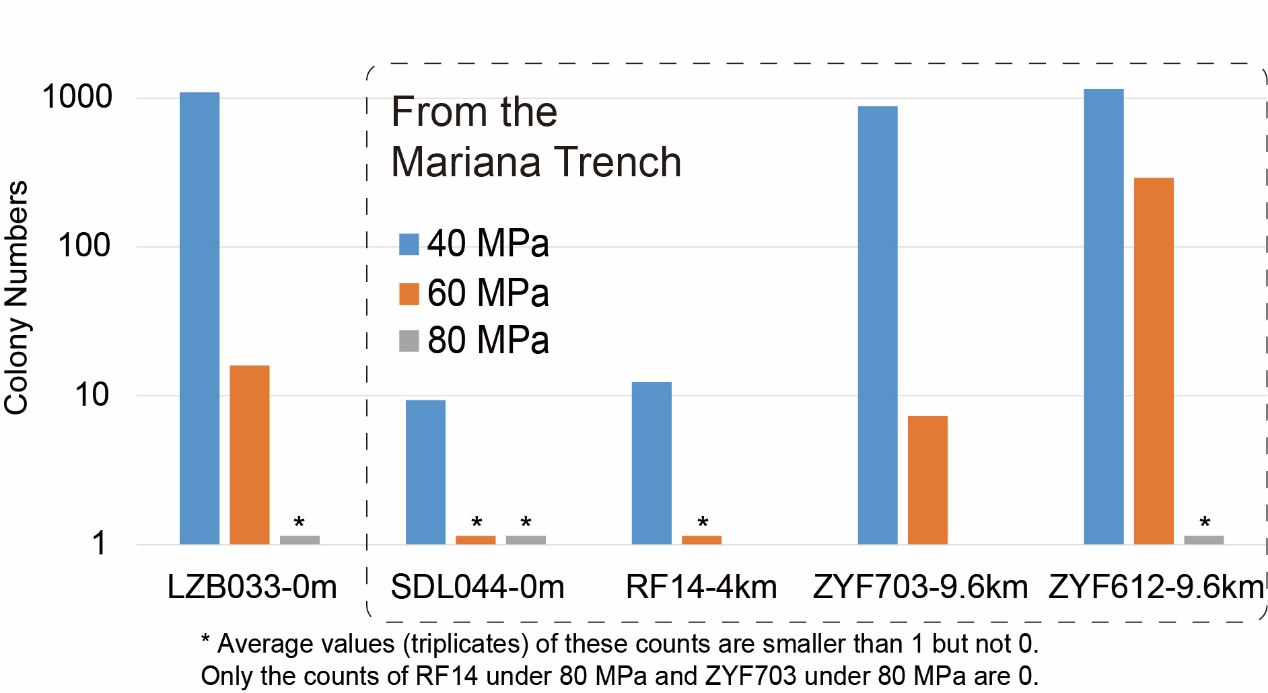


**Figure S1.** Average colony counts of the five *L. aggregata* strains after cultivating under 40, 60 and 80 MPa (equal to 4,000, 6,000 and 8,000 depth). After incubating the strains in 2.5 mL syringes (3 syringes each strains as triplicates) in high pressure reactors for 2 weeks, the colony numbers were counted based on spread plate method cultivating under room temperature for two days. Generally all the strains could resist 40 MPa and deactivated (or grow extremely slowly) under 80 MPa. The strain ZYF612 from 9.6 km shows the highest resistance against high hydrostatic pressure under 60 MPa.


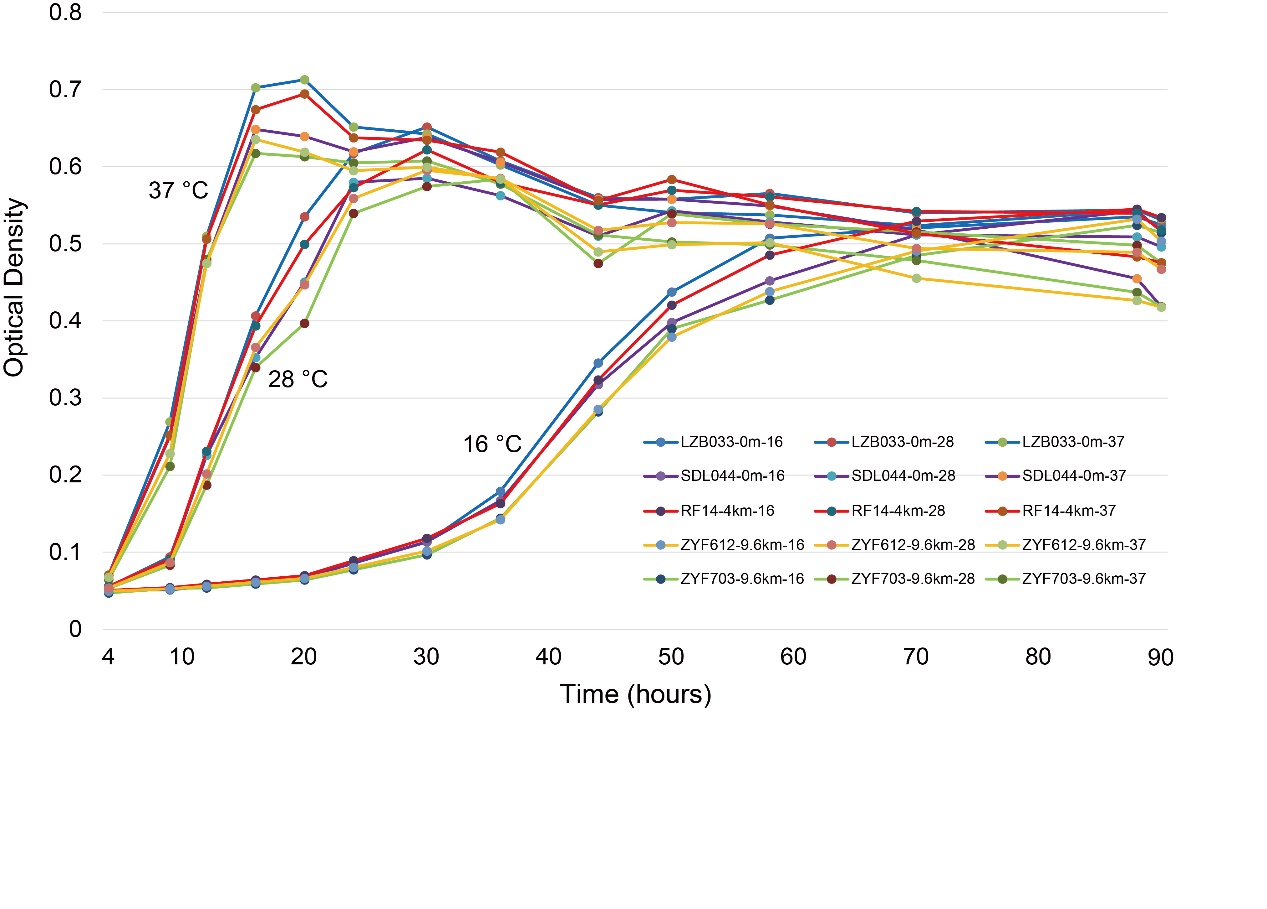


**Figure S2**. Growth curves of the five *L. aggregata* strains after cultivating under atmospheric pressure and different temperatures for 90 hours. All the five strains were also cultivated under 4 °C, but the growth could barely be observed and thus is not shown in the figure.


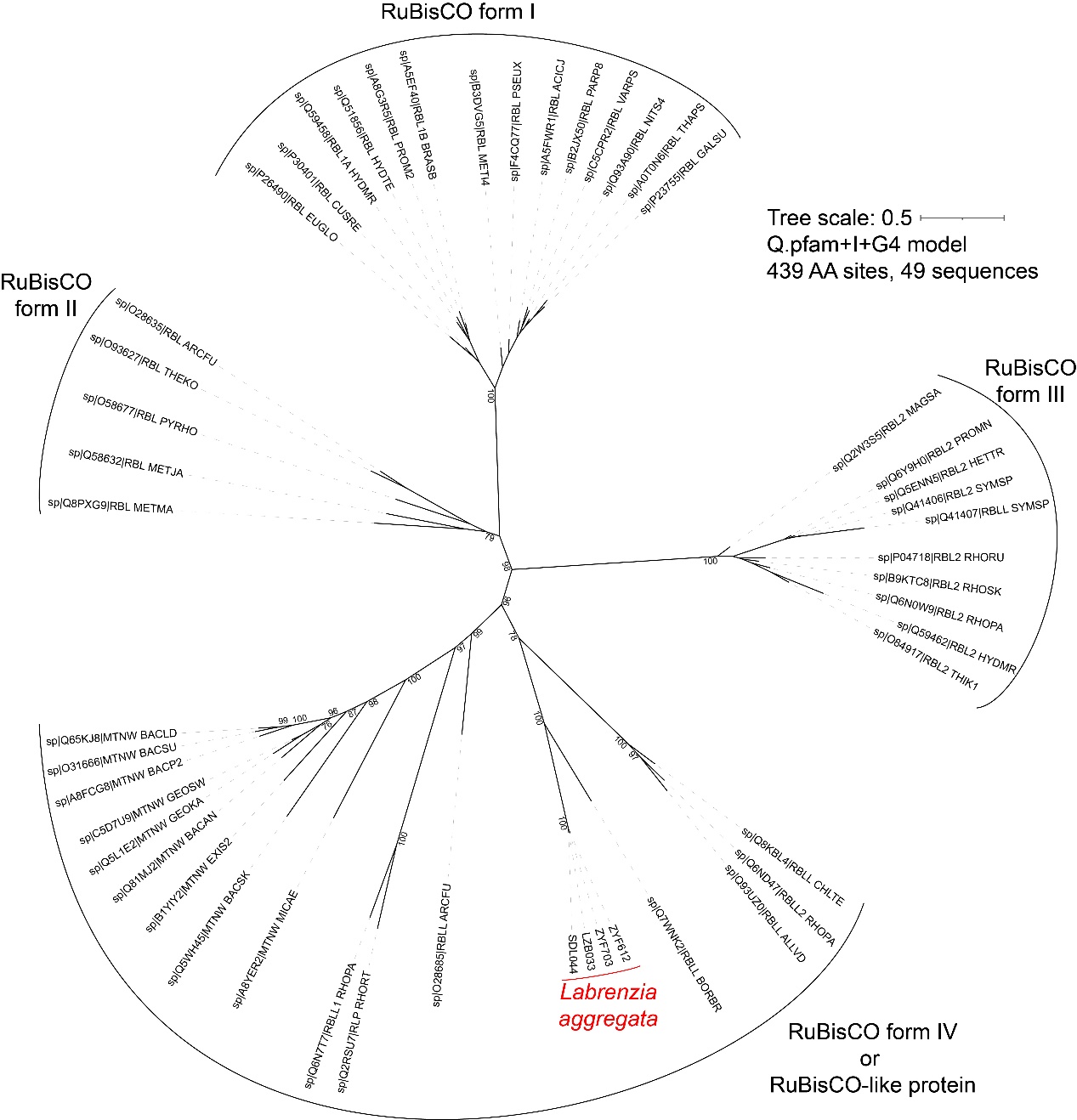


**Figure S3.** Phylogenetic tree of the *rbcL* genes in *L. aggregata*. All the four sequences belong to form IV RuBisCO, which are incapable of carbon fixation like the classical form I RuBisCO in Calvin cycle. The name of reference sequences are from UniProt database.


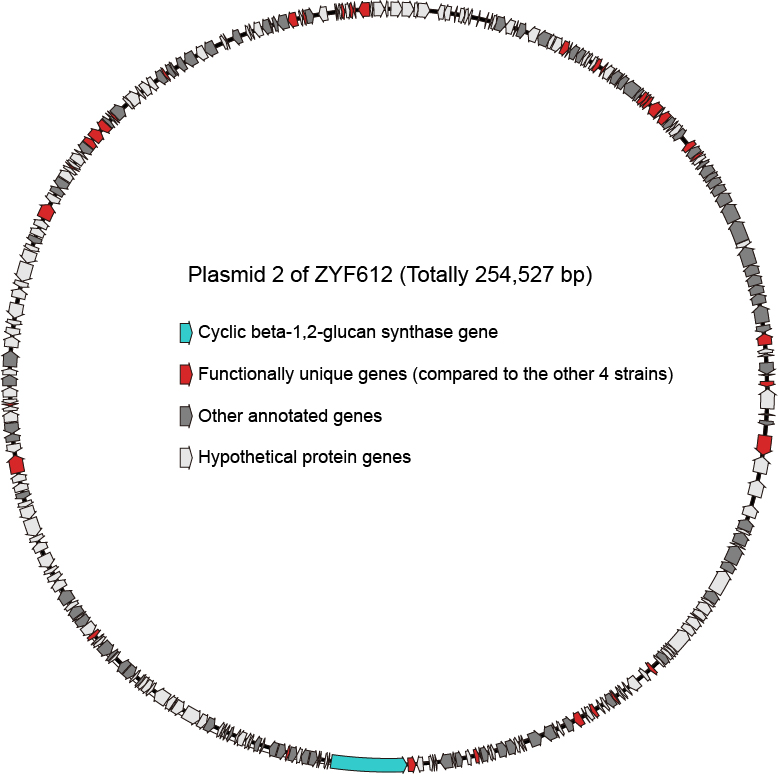


**Figure S4.** Cyclic beta-1,2-glucan synthase gene in the plasmid 2 of ZYF612 (ZYF612P2). Functionally unique genes are determined based on the results of Figure 2. The lengths of the arrows are in scale with gene lengths.


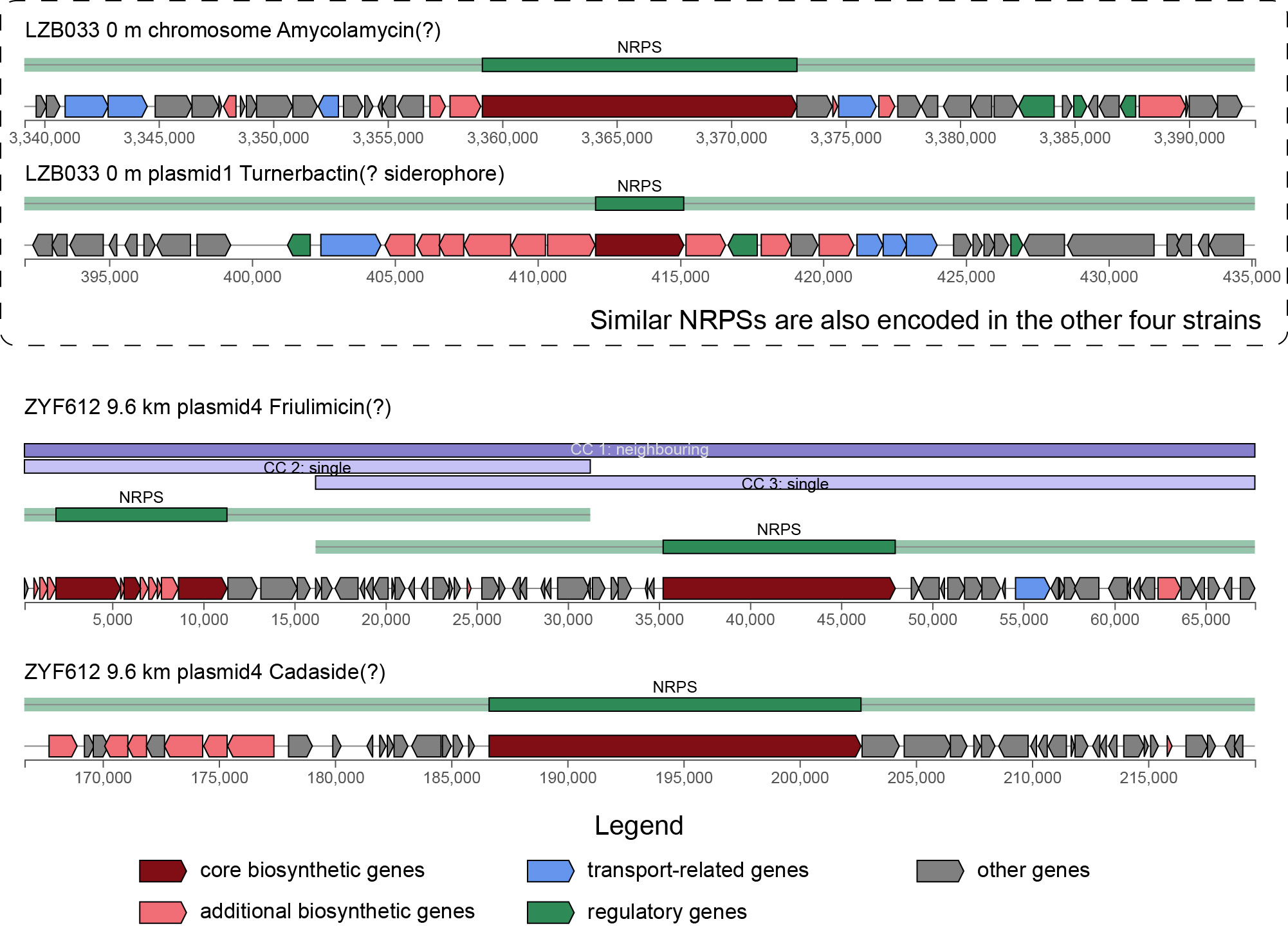


**Figure S5.** NRPS and related genes in the five strains. The upper two NRPS gene clusters are shared by all the five strains, while the lower ones are specific in the strain ZYF612. Regions containing NRPS and related genes were shown. The length of the arrows is in scale with the genes. All these NRPS gene clusters are predicted with rough functions only (antibiotic or siderophore) and their true products require future studies.


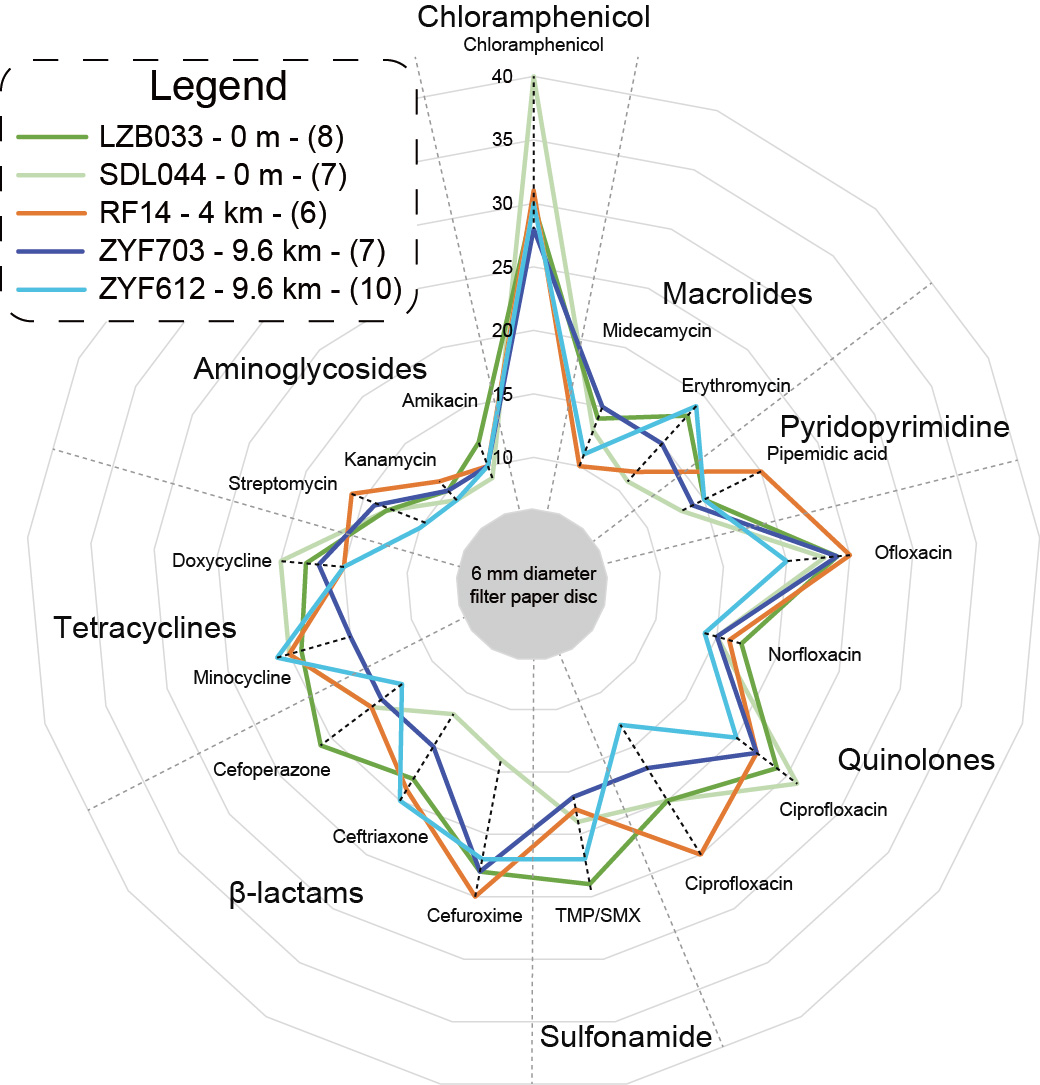


**Figure S6.** Antibiotics disk diffusion test results of the five strains. The smaller the inhibition zone is the relatively stronger antibiotic resistance the strain has. The numbers in the brackets indicate the hit numbers of genes against SARG database by blastp with identities higher than 50%. These antibiotics were selected according to a pre-test confirming their effectiveness.

**Table S1.** Average nucleotide identities (written before [ ]) and aligned nucleotides percentages (written in [ ]) between the *L. aggregata* strains. Genomes were aligned based on MUMmer and their identities and aligned nucleotides percentages were calculated in JspeciesWS with default settings.

|  | RMAR6-6 | LZB033 (0 m) | SDL044 (0 m) | RF14 (4 km) | ZYF703 (9.6 km) | ZYF612 (9.6 km) |
| --- | --- | --- | --- | --- | --- | --- |
| RMAR6-6 | * | 97.89 [90.42] | 97.68 [91.25] | 97.61 [88.43] | 97.88 [90.11] | 97.58 [90.95] |
| LZB033 (0 m) | 97.89 [93.21] | * | 97.64 [94.53] | 97.59 [91.65] | 98.12 [95.87] | 97.60 [93.47] |
| SDL044 (0 m) | 97.68 [93.45] | 97.64 [93.97] | * | 97.81 [91.47] | 97.62 [94.02] | 97.61 [93.86] |
| RF14 (4 km) | 97.61 [90.16] | 97.59 [90.63] | 97.81 [91.37] | * | 97.53 [91.93] | 97.59 [90.63] |
| ZYF703 (9.6 km) | 97.88 [89.94] | 98.12 [93.00] | 97.62 [91.69] | 97.53 [89.91] | * | 97.55 [91.60] |
| ZYF612 (9.6 km) | 97.58 [87.74] | 97.60 [87.62] | 97.61 [88.72] | 97.59 [86.15] | 97.55 [88.93] | * |

**Table S2.** Estimation of plasmids copy numbers based on Illumina reads mapping. The plasmids of ZYF612 and RF14 are likely to be multiple copies.

|  | ZYF612 (9.6 km) | ZYF703 (9.6 km) | RF14 (4 km) | SDL044 (0 m) | LZB033 (0 m) |
| --- | --- | --- | --- | --- | --- |
|  | Sequencing Depth Ratio (Plasmid_Depth/Chromosome_Depth) | | | | |
| Plasmids | 1.319207043 | 0.769197964 | 4.427205631 | 0.706358578 | 0.860495901 |
|  | 2.819697666 | 0.326351082 | 5.842477092 | 0.31433852 | 0.609945101 |
|  | 3.950539686 | 0.434734497 | 17.60725728 | 0.36680654 |  |
|  | 3.416851365 |  | 11.96768656 | 0.309059752 |  |
|  | Estimates of Plasmids Copy Numbers | | | | |
| Plasmid 1 | 2 | 1 | 5 | 1 | 1 |
| Plasmid 2 | 3 | 1 | 6 | 1 | 1 |
| Plasmid 3 | 4 | 1 | 18 | 1 |  |
| Plasmid 4 | 4 |  | 12 | 1 |  |

**Table S3**. The numbers of different gene origins in the five *L. aggregata* strains according to the most similar annotation by EggNOG database v5.0. *L. aggregata* belongs to domain: *Bacteria*, phylum: *Proteobacteria*, class: *Alphaproteobacteria*, order: *Rhodobacterales*, family: *Rhodobacteraceae* and genus: *Labrenzia*. These taxonomy ranks are labelled in red fonts. The gene numbers larger than 10 and 1.5 fold larger than average were labelled in yellow, which could contribute to the extra CDSs in these genomes.

| Gene Classification | LZB033 | SDL044 | RF14 | ZYF703 | ZYF612 |
| --- | --- | --- | --- | --- | --- |
| Alphaproteobacteria | 4812 | 4825 | 4860 | 4915 | 5017 |
| Bacteria | 147 | 151 | 142 | 150 | 168 |
| Rhizobiaceae | 109 | 148 | 147 | 117 | 162 |
| Phyllobacteriaceae | 116 | 112 | 118 | 120 | 99 |
| Proteobacteria | 83 | 86 | 78 | 83 | 91 |
| Rhodospirillales | 43 | 33 | 26 | 36 | 49 |
| Bradyrhizobiaceae | 23 | 25 | 35 | 29 | 49 |
| Paracoccus | 22 | 25 | 23 | 23 | 25 |
| unclassified Alphaproteobacteria | 15 | 20 | 22 | 19 | 25 |
| Gammaproteobacteria | 18 | 17 | 19 | 19 | 23 |
| Oceanicola | 10 | 6 | 30 | 35 | 8 |
| Sphingomonadales | 12 | 9 | 10 | 13 | 35 |
| Thioclava | 13 | 11 | 16 | 7 | 15 |
| Methylobacteriaceae | 11 | 12 | 11 | 13 | 11 |
| Ruegeria | 9 | 8 | 13 | 13 | 11 |
| Roseobacter | 10 | 13 | 11 | 9 | 10 |
| Roseovarius | 11 | 12 | 7 | 12 | 10 |
| Brucellaceae | 7 | 10 | 8 | 15 | 12 |
| Sulfitobacter | 5 | 11 | 9 | 8 | 13 |
| Aurantimonadaceae | 8 | 7 | 4 | 12 | 11 |
| Hyphomicrobiaceae | 7 | 6 | 10 | 7 | 8 |
| Hyphomonadaceae | 6 | 7 | 8 | 5 | 4 |
| Burkholderiaceae | 3 | 5 | 4 | 8 | 8 |
| Actinobacteria | 3 | 5 | 4 | 5 | 10 |
| Xanthobacteraceae | 5 | 7 | 6 | 7 | 2 |
| Rhodobiaceae | 7 | 4 | 8 | 6 | 2 |
| Oceanospirillales | 4 | 6 | 6 | 6 | 4 |
| Phaeobacter | 4 | 4 | 3 | 3 | 9 |
| Vibrionales | 4 | 5 | 5 | 4 | 5 |
| Beijerinckiaceae | 5 | 4 | 5 | 5 | 3 |
| Planctomycetes | 4 | 4 | 4 | 4 | 4 |
| Leisingera | 2 | 3 | 10 | 1 | 4 |
| Betaproteobacteria | 3 | 5 | 1 | 5 | 5 |
| Methylocystaceae | 2 | 1 | 9 | 3 | 4 |
| Comamonadaceae | 3 | 3 | 5 | 4 | 2 |
| Roseivivax | 3 | 3 | 1 | 3 | 5 |
| Xanthomonadales | 2 | 3 | 1 | 3 | 5 |
| Bartonellaceae | 1 | 3 | 4 | 3 | 3 |
| Rhodovulum | 3 | 2 | 2 | 3 | 2 |
| Moraxellaceae | 3 | 2 | 3 | 1 | 3 |
| Halobacteria | 2 | 2 | 3 | 2 | 2 |
| Alteromonadaceae | 3 | 2 | 2 | 3 | 1 |
| Legionellales | 2 | 2 | 3 | 2 | 2 |
| unclassified Rhodobacteraceae | 1 | 1 | 3 | 2 | 3 |
| Caulobacterales | 1 | 1 | 3 | 1 | 4 |
| Desulfovibrionales | 1 | 1 | 2 | 3 | 2 |
| Nostocales | 1 | 1 | 2 | 1 | 4 |
| Bacilli | 1 | 3 | 1 | 1 | 3 |
| unclassified Burkholderiales | 1 | 1 | 3 | 2 | 1 |
| Cytophagia | 2 | 1 | 1 | 2 | 2 |
| Deltaproteobacteria | 1 | 2 | 1 | 1 | 3 |
| Nocardiaceae | 1 | 1 | 1 | 2 | 2 |
| Oscillatoriales | 2 | 1 | 1 | 2 | 1 |
| delta/epsilon subdivisions | 1 | 1 | 1 | 0 | 4 |
| Shewanellaceae | 2 | 1 | 1 | 1 | 2 |
| Oxalobacteraceae | 1 | 2 | 1 | 1 | 1 |
| Cyanobacteria | 2 | 1 | 1 | 1 | 1 |
| Alteromonadales genera incertae sedis | 1 | 1 | 1 | 1 | 1 |
| Desulfobacterales | 3 | 0 | 1 | 0 | 1 |
| Alcaligenaceae | 2 | 1 | 1 | 1 | 0 |
| Bacillus | 3 | 0 | 1 | 1 | 0 |
| Epsilonproteobacteria | 1 | 1 | 1 | 1 | 1 |
| Nitrosomonadales | 0 | 1 | 1 | 1 | 2 |
| Providencia | 1 | 1 | 1 | 1 | 1 |
| Acidithiobacillales | 0 | 0 | 0 | 0 | 4 |
| Thiotrichales | 0 | 2 | 1 | 0 | 1 |
| Ruminococcaceae | 0 | 1 | 0 | 1 | 2 |
| Planococcaceae | 0 | 1 | 1 | 0 | 1 |
| Synechococcus | 1 | 0 | 0 | 0 | 2 |
| Pseudonocardiales | 2 | 0 | 0 | 0 | 1 |
| Pleurocapsales | 0 | 1 | 1 | 0 | 1 |
| Chromatiales | 2 | 0 | 0 | 1 | 0 |
| Bacteroidia | 1 | 1 | 1 | 0 | 0 |
| Bacillariophyta | 1 | 0 | 0 | 1 | 0 |
| Rubrobacteria | 1 | 0 | 0 | 0 | 1 |
| unclassified Gammaproteobacteria | 1 | 1 | 0 | 0 | 0 |
| Clostridia | 0 | 0 | 0 | 1 | 1 |
| Frankiales | 0 | 0 | 2 | 0 | 0 |
| Paenibacillaceae | 0 | 0 | 0 | 0 | 2 |
| Aquimarina | 0 | 0 | 0 | 0 | 1 |
| Rhodocyclales | 0 | 0 | 1 | 0 | 0 |
| Flavobacteriia | 1 | 0 | 0 | 0 | 0 |
| Pseudomonas aeruginosa group | 1 | 0 | 0 | 0 | 0 |
| Erysipelotrichia | 1 | 0 | 0 | 0 | 0 |
| Halanaerobiales | 1 | 0 | 0 | 0 | 0 |
| Carnobacteriaceae | 0 | 0 | 1 | 0 | 0 |
| Rickettsiales | 0 | 0 | 1 | 0 | 0 |
| Myoviridae | 0 | 0 | 0 | 0 | 1 |
| Microbacteriaceae | 0 | 0 | 0 | 0 | 1 |
| Podoviridae | 0 | 0 | 0 | 1 | 0 |
| Rhodobacter | 0 | 1 | 0 | 0 | 0 |
| Methanococci | 0 | 0 | 0 | 0 | 1 |
| Dermabacteraceae | 0 | 1 | 0 | 0 | 0 |
| Siphoviridae | 0 | 1 | 0 | 0 | 0 |
| Micromonosporales | 0 | 0 | 0 | 0 | 1 |
| Clostridiaceae | 1 | 0 | 0 | 0 | 0 |
| Euryarchaeota | 1 | 0 | 0 | 0 | 0 |
| Micrococcaceae | 0 | 0 | 0 | 1 | 0 |
| Streptosporangiales | 0 | 1 | 0 | 0 | 0 |
| Tatumella | 0 | 0 | 0 | 0 | 1 |
| Negativicutes | 1 | 0 | 0 | 0 | 0 |

**Table S4.** Prophages predicted in the five *L. aggregata* strains by PHASTST with default settings. Intact prophages are labelled in red.

| Strain | Depth (m) | Chromosome/  Plasmids | Prophage  Length | Completeness | # Total  Proteins | GC % | Region Position | Prophage Classification and NCBI Accession |
| --- | --- | --- | --- | --- | --- | --- | --- | --- |
| LZB033 | 0 | chromosome | 12.6Kb | incomplete | 11 | 60.52% | 477972-490642 | PHAGE_Klebsi_ST16_OXA48phi5.4_NC_049450(1) |
| SDL044 | 0 | chromosome | 12.6Kb | incomplete | 12 | 60.71% | 443050-455691 | PHAGE_Bacill_Staley_NC_022767(1) |
| RF14 | 4,000 | chromosome | 30.4Kb | questionable | 46 | 59.79% | 5641788-5672203 | PHAGE_Rhizob_16_3_NC_011103(12) |
| RF14 | 4,000 | RF14P2 | 6.4Kb | questionable | 10 | 57.88% | 535-7004 | PHAGE_Stx2_c_Stx2a_F451_NC_049924(3) |
| RF14 | 4,000 | RF14P2 | 9.3Kb | incomplete | 9 | 59.51% | 32679-42063 | PHAGE_Escher_SH2026Stx1_NC_049919(3) |
| RF14 | 4,000 | RF14P2 | 6.4Kb | questionable | 10 | 57.85% | 226825-233318 | PHAGE_Stx2_c_Stx2a_F451_NC_049924(3) |
| RF14 | 4,000 | RF14P3 | 25.8Kb | incomplete | 13 | 57.11% | 24201-50033 | PHAGE_Stx2_c_Stx2a_F451_NC_049924(3) |
| RF14 | 4,000 | RF14P4 | 29Kb | incomplete | 14 | 56.27% | 2718-31774 | PHAGE_Pectob_CBB_NC_041878(2) |
| RF14 | 4,000 | RF14P4 | 27.5Kb | incomplete | 17 | 55.77% | 70149-97673 | PHAGE_Pectob_CBB_NC_041878(2) |
| ZYF703 | 9,600 | chromosome | 54.8Kb | intact | 55 | 55.99% | 3706494-3761317 | PHAGE_Aurant_AmM_1_NC_027334(15) |
| ZYF703 | 9,600 | chromosome | 27Kb | incomplete | 33 | 60.51% | 4224273-4251274 | PHAGE_Rhodob_RcRhea_NC_028954(8) |
| ZYF612 | 9,600 | chromosome | 46.4Kb | intact | 44 | 59.17% | 5374920-5421392 | PHAGE_Rhodob_RC1_NC_020839(14) |
| ZYF612 | 9,600 | ZYF612P2 | 7.7Kb | incomplete | 9 | 57.39% | 16345-24114 | PHAGE_Gordon_Schwabeltier_NC_031255(1) |
| ZYF612 | 9,600 | ZYF612P3 | 8.8Kb | incomplete | 8 | 55.49% | 91037-99845 | PHAGE_Pectob_CBB_NC_041878(2) |
| ZYF612 | 9,600 | ZYF612P4 | 15.8Kb | intact | 26 | 54.53% | 16167-32040 | PHAGE_Paenib_Tripp_NC_028930(2) |
| ZYF612 | 9,600 | ZYF612P4 | 13.3Kb | questionable | 17 | 58.21% | 48857-62206 | PHAGE_Stx2_c_Stx2a_F451_NC_049924(3) |
| ZYF612 | 9,600 | ZYF612P4 | 7.2Kb | incomplete | 9 | 53.86% | 117998-125217 | PHAGE_Escher_SH2026Stx1_NC_049919(2) |
| ZYF612 | 9,600 | ZYF612P4 | 11.5Kb | intact | 20 | 56.60% | 207505-219075 | PHAGE_Stx2_c_1717_NC_011357(2) |
